# Efficacy of High-Dose Mycophenolate Mofetil in Multitarget Therapy for Lupus Nephritis: Two Consecutive Case Reports

**DOI:** 10.7759/cureus.6834

**Published:** 2020-01-31

**Authors:** Yoshitaka Furuto, Mariko Kawamura, Akio Namikawa, Hiroko Takahashi, Yuko Shibuya

**Affiliations:** 1 Hypertension and Nephrology, NTT Medical Centre Tokyo, Tokyo, JPN

**Keywords:** lupus nephritis, multitarget therapy, mycophenolate mofetil, tacrolimus, steroids, induction therapy

## Abstract

The complete remission rate for lupus nephritis (LN) is higher with multitarget therapy (MT) using tacrolimus (TAC), mycophenolate mofetil (MMF), and steroids than with steroid plus cyclophosphamide co-therapy. MT is also considered highly safe and is used to treat refractory LN. During MT, MMF is usually administered at a dose of 1 g/day similar to conventional MT; however, it remains unclear whether this is the optimal dose of MMF for Japanese patients, especially those refractories to conventional MT. We report two consecutive cases of refractory LN with conventional MT, case 1 was a 48-year-old woman with LN III (A) and nephrotic syndrome, and Case 2 was a 20-year-old man with LN IV-S (A), nephrotic syndrome, and acute kidney injury. LN was diagnosed by kidney biopsy. Because both these patients were refractory to conventional MT treatment (MMF at a dose of 1.0 g/day) for more than six months, MMF doses of 2.5 and 1.5-2.0 g/day were used as part of MT for cases 1 and 2, respectively. Increasing the MMF dose in MT to 1.5-2.5 g/day without increasing the steroid dose led to complete remission, without any recurrence, and allowed administration of a lower dose of a steroid such as prednisolone (5.5 ± 1.5 mg/day) 18 months after the MMF dose increase. The mean number of days from the start of the higher MMF dose of 1.5-2.5 g/day in MT to complete remission was 129.5 ± 10.5 days. Moreover, lymphopenia, hypogammaglobulinemia, gastrointestinal disturbances, or any infections were not observed as adverse events after increasing the MMF dose in MT. Thus, increasing MMF dose while maintaining the steroid dose in MT may induce complete remission; this will minimize the use of steroids in Japanese patients with refractory LN in conventional MT.

## Introduction

Kidney injury occurs in more than 60% of systemic lupus erythematosus (SLE) cases. As kidney injury contributes to morbidity and mortality, inducing remission of lupus nephritis (LN) is important [1]. Mycophenolate mofetil (MMF) is widely recognized as a remission-inducing drug for LN and was approved in May 2016 for the treatment of SLE in Japan. According to the Kidney Disease: Improving Global Outcomes (KDIGO) and American College of Rheumatology (ACR) guidelines, coadministration of high-dose steroids and either cyclophosphamide (CYC) or MMF is the recommended remission-inducing therapy for the initial treatment of stage III and IV LN [1-2]. MMF exhibits the same efficacy as does CYC, the conventional standard therapeutic agent used to treat severe LN [3]. MMF is also well-tolerated and is a recommended treatment option for adult Asian patients [3-5]. MMF is associated with fewer adverse events, such as serious infections, alopecia, and amenorrhea, and is thus considered superior to CYC [4]. MMF treatment is cost-effective, with a reduced number of hospital admissions due to infection and is associated with higher quality-of-life scores than CYC is [6-7]. In addition, treatment with MMF results in a high complete remission rate, and the drug shows a good safety profile [8]. The calcineurin inhibitor tacrolimus (TAC) is another remission-inducing drug, and combination therapy with TAC and steroids has been found to be successful [9].

A study in Chinese patients has reported that the use of a triple-target therapy consisting of a steroid, TAC, and MMF may result in a higher complete remission rate than when steroids and injected CYC are used (65% vs. 15%) [10]. Multitarget therapy (MT), including a glucocorticoid (GC), which targets multiple immunity mechanisms through the combined use of immunosuppressants, is designed to achieve a synergistic effect using calcineurin inhibitors (T-cell suppressants) and purine metabolism inhibitors (B-cell suppressants), allowing minimal GC dose administration [10]. This MT is used for clinically severe and highly pathologically active cases, with high proteinuria levels, which require a considerable time to achieve remission [11]. The use of combinations of immunosuppressants in MT consisting of GC+TAC+MMF has been reported recently, it has several merits, including a high remission rate, low nephrotoxicity, and a short time to remission [10-11].

Previous studies of the MMF dose used in MT to treat Chinese patients with stage III, IV, and V LN reported doses of 1.0 g/day and 0.75-1.0 g/day [10-11]. In these previous studies of MT in Asian patients, an MMF dose of 1 g/day was administered; however, it is unknown whether this is the optimal dose for Japanese patients. Here, we report two consecutive cases in which MT was successfully used to induce complete remission of LN that was refractory to conventional MT by increasing the MMF dose to 1.5-2.5 g/day without increasing the steroid dose.

## Case presentation

Informed consent was obtained from all patients for the participation and publication of these case reports.

Case 1

A 48-year-old woman presented with the chief complaint of edema. She had subjective symptoms of fever and general malaise in July 2010, followed by an apparent loss of appetite in early August. In mid-August, she was diagnosed with nephrotic syndrome and was admitted to the hospital.

The patient had sinusitis at the age of 46 years. The family history or history of allergy was non-significant. She was a housewife, an occasional drinker, and a tobacco user (10 cigarettes/day for 28 years). The physical findings at admission were as follows: height, 159 cm; body weight (BW), 61.5 kg; body mass index (BMI), 24.3; blood pressure (BP), 124/74 mmHg; heart rate (HR), 57/min; body temperature (BT), 36.6 °C; saturation of percutaneous oxygen (SpO2), 96%; mental state, conscious and alert. She had palpebral edema but no pallor, sinus pressure pain, significant oral cavity findings, swellings of cervical lymph nodes, cardiac murmur, or abnormal pulmonary sounds. Her abdomen was flat and soft, although slight distension was present. She also had lower extremity edema but no joint pain. There was no photosensitivity or positive dermatological or neurological findings.

**Figure 1:**
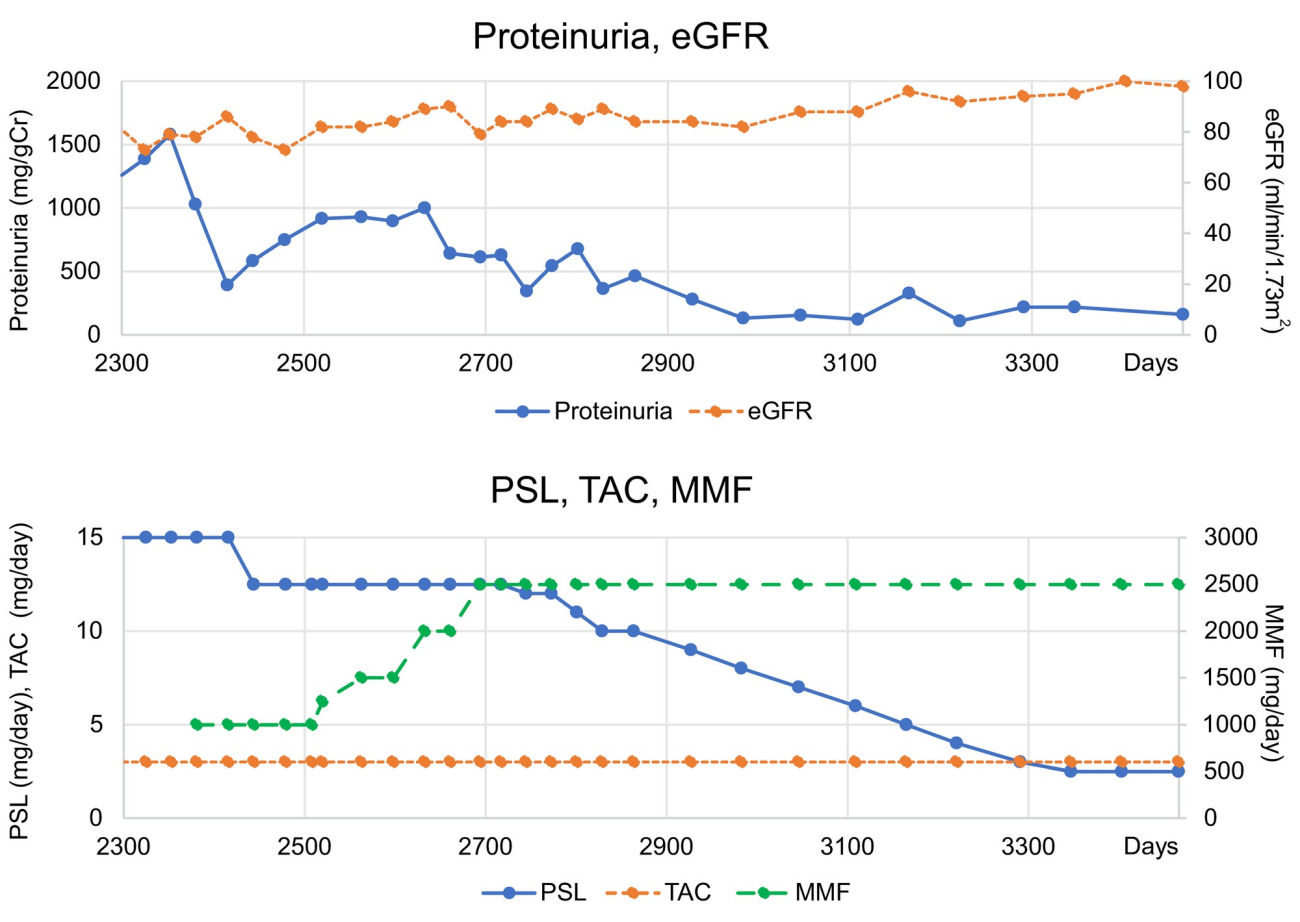
Case 1 data. Time courses of proteinuria and eGFR (upper panel) and PSL, TAC, and MMF dosing (lower panel). eGFR: Estimated Glomerular Filtration Rate; MMF: Mycophenolate Mofetil; PSL: Prednisolone; TAC: Tacrolimus.

The laboratory findings are presented in Table 1, and the therapeutic progress is shown in Figure 1. Based on the diagnostic protocol from the Systemic Lupus International Collaborating Clinics (SLICC) SLE classification guidelines of 2012, the following clinical and immunological findings met the diagnostic criteria for SLE: proteinuria ≥ 0.5 g/day; WBC < 4000; lymphocyte count < 1000; positive antinuclear antibody; positive anti-Smith antibody; and hypocomplementemia [12]. The SLE disease activity index (SLEDAI) score was 15. A kidney biopsy was performed, and the patient was diagnosed with LN III (A) based on the International Society of Nephrology/Renal Pathology Society (ISN/RPS) criteria.

**Table 1:** Laboratory data of the patients on admission HFP: High-Power Field; TP: Total Protein; UA: Uric Acid; BUN: Blood Urea Nitrogen; Cr: Creatinine; eGFR, Estimated Glomerular Filtration Rate; AST: Aspartate Aminotransferase; ALT: Alanine Aminotransferase; LDH: Lactate Dehydrogenase; CRP: C-reactive Protein; TC: Total Cholesterol; LDL-C: Low-Density Lipoprotein Cholesterol; C3: Complement 3; C4: complement 4; CH50: complement 50; Ab: antibody; dsDNA: double-stranded DNA; Sm: Smith; RNP: ribonucleoprotein; CL-β2GP: cardiolipin beta 2 glycoprotein.

Analysis/Test	Case 1	Case 2
Protein	3+	3+
Occult blood	±	3+
Red blood cells (/HPF)	1–4	20–29
Protein/creatinine ratio (g/g Cr)	8.6	2.6
White blood cells (/μL)	3200	2700
Neutrophils	2100 (66.8%)	2092 (77.5%)
Lymphocytes	710 (22.5%)	324 (12.0%)
Monocytes	7.9%	8.5%
Eosinophils	2.5%	0%
Red blood cells (/μL)	423 × 10^4^	396 × 10^4^
Hemoglobin (g/dL)	12.8	11.3
Hematocrit	37.8%	34.5%
Platelets (/μL)	17.8 × 10^4^	5.4 × 10^4^
TP (g/dL)	4.7	4.7
Albumin (g/dL)	1.2	1.9
UA (mg/dL)	4.4	9.7
BUN (mg/dL)	12.9	74.3
Cr (mg/dL)	0.53	2.45
eGFR (mL/min/1.73 m^2^)	95	31
AST (U/L)	18	782
ALT (U/L)	15	575
LDH (U/L)	215	614
CRP (mg/dL)	0.3	5.2
TC (mg/dL)	379	238
LDL-C (mg/dL)	255	92
IgG (mg/dL)	1455	1172
C3 (mg/dL)	52	24
C4 (mg/dL)	22.3	2.2
CH50 (U/mL)	5	5
Antinuclear Ab	1:1,280	1:1,280
Anti-dsDNA Ab (IU/mL)	11.2 (−)	380 (+)
Anti-Sm Ab (U/mL)	101.6 (+)	(−)
Anti-RNP Ab (U/mL)	169.3 (+)	(−)
Anti-CL-β2GP1 Ab	(−)	(−)

 

Treatment was initiated with 50 mg/day prednisolone (PSL). On day 14, the proteinuria level indicated remission because the estimated glomerular filtration rate (eGFR), hypocomplementemia, and the anti-double-stranded (ds) DNA antibody level improved, while PSL was gradually decreased. On day 36, the PSL dose was 35 mg/day, and the patient was discharged. The PSL dose was gradually decreased to 15 mg/day by day 140; however, the patient experienced subsequent recurrence of proteinuria. On day 260, the proteinuria level was elevated to 2581 mg/g creatinine (Cr), and the PSL dose was increased to 40 mg/day. From day 260 to day 2352, MZR 100-150 mg/day with PSL, TAC 2-3 mg/day with PSL, or combination therapy of MZR and TAC with PSL was administered. However, despite a temporary improvement in proteinuria, remission was difficult to maintain, and the proteinuria level remained at approximately 1,000 mg/g Cr. Therefore, it was impossible to reduce the PSL dose to <15 mg/day. Thus, on day 2353, the patient was started on a course of MT consisting of PSL 15 mg/day + TAC 3 mg/day + MMF 1.0 g/day. With the MMF dose maintained at 1.0 g/day for 6 months, proteinuria levels remained above 0.5 g/g Cr and showed no remission, and improvement in proteinuria was poor; therefore, we considered this case as refractory LN. The PSL dose could not be reduced to <12.5 mg/day, indicating that a satisfactory outcome was not achieved. Therefore, the MMF dose was gradually increased from 1 to 2.5 g/day without increasing steroid dose in MT by day 2695. On day 2829, the proteinuria level improved to <0.5 g/day, indicating complete remission. Subsequently, the PSL dose was gradually reduced to 2.5 mg/day on day 3347. Thus, both induction and maintenance of complete remission were achieved. We believe that it is effective to increase the MMF dose to 2.5 g/day when using MT consisting of PSL+TAC+MMF for LN III (A). Complete remission was achieved 140 days after increasing the MMF dose to 2.5 g/day. No adverse events were observed.

 

Case 2

A 20-year-old man presented with the chief complaint of malaise. He had subjective symptoms of poor appetite and systemic malaise from February 2017 and diarrhea from March 2017. The patient was diagnosed with pancytopenia, acute kidney injury, and hepatic dysfunction and was admitted on an emergency basis. The past medical history, family history, and history of allergy were non-significant. The patient was a student and non-smoker and had no history of alcoholic beverage intake, overseas travel, or casual sex.

**Figure 2:**
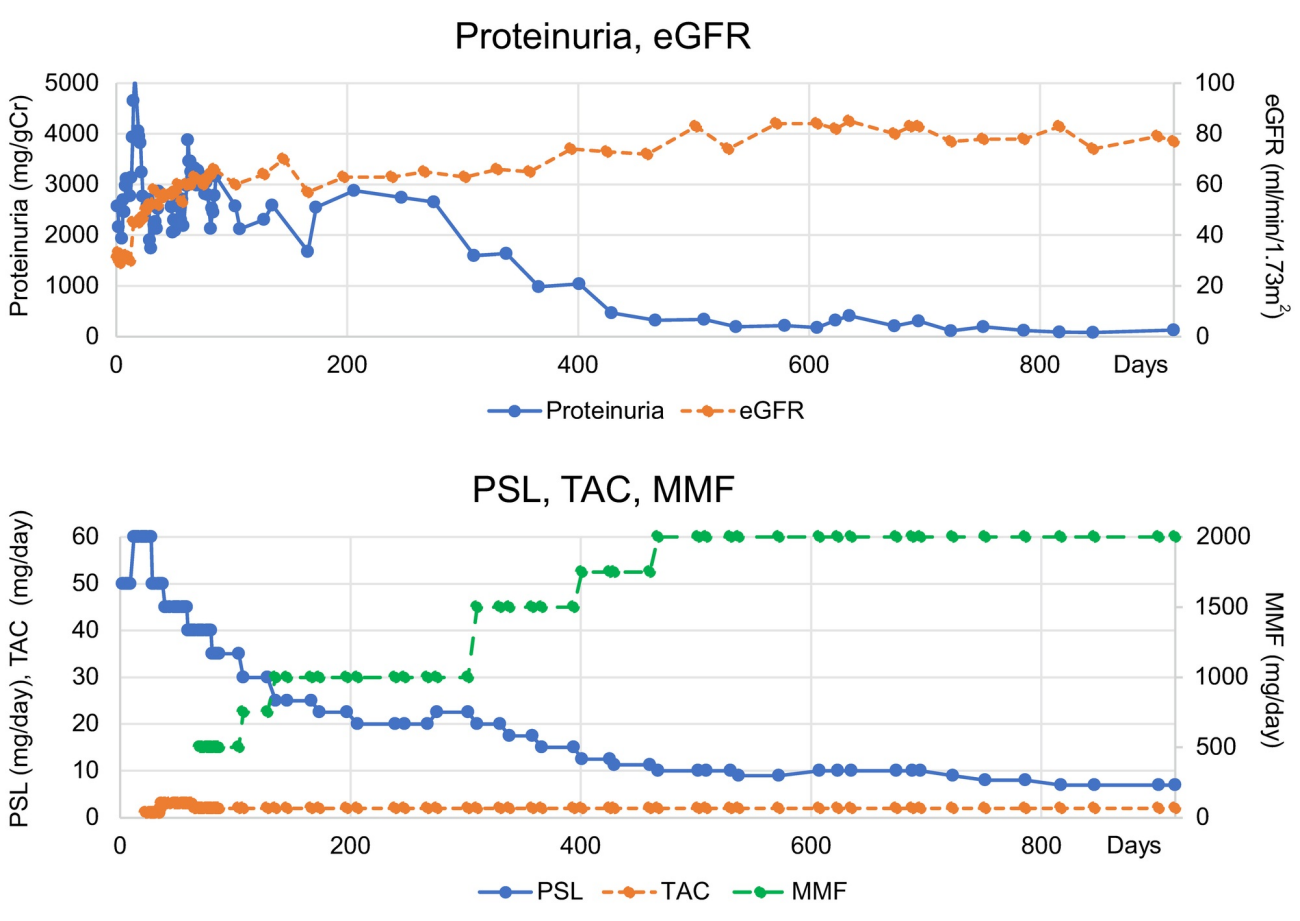
Case 2 data. Time courses of proteinuria and eGFR (upper panel) and PSL, TAC, and MMF dosing (lower panel). eGFR: Estimated Glomerular Filtration Rate; MMF: Mycophenolate Mofetil; PSL: Prednisolone; TAC: Tacrolimus

The physical findings at admission were as follows: height, 173.0 cm; BW, 60.7 kg; BMI, 24.3; BP, 108/77 mmHg; HR, 104/min; BT, 36.5 °C; SpO2, 99%; mental state, conscious and alert; the presence of pallor and an oral aphthous ulcer. There was no sinus pressure pain, superficial lymph node swelling, cardiac murmur, or abnormal pulmonary sounds. The abdomen was flat and soft (no rebound tenderness). Murphy signs, hepatosplenomegaly, and costovertebral angle tenderness were not observed. There was no lower extremity edema and no photosensitivity or positive significant joint findings, dermatological findings, or neurological findings. The laboratory findings are shown in Table 1, and the therapeutic progress is shown in Figure 2. Based on the diagnostic protocol from the SLICC SLE classification guidelines of 2012, the following clinical and immunological findings met the diagnostic criteria for SLE: proteinuria ≥ 0.5 g/day, oral ulcer, hemolytic anemia, leukopenia, lymphopenia, thrombocytopenia, positive anti-nuclear antibody, positive anti-dsDNA antibody, and hypocomplementemia [12]. His SLEDAI score was 20, which also met the diagnostic standard for SLE. A kidney biopsy was performed, and the patient was diagnosed with LN IV-S (A) based on the ISN/RPS criteria. Liver dysfunction was observed, while tests for viral infection and other immune diseases were negative. The patient was diagnosed with SLE with liver dysfunction.

On day 2, the patient underwent treatment with PSL 50 mg/day, which was increased to 60 mg/day on day 12. He also underwent steroid pulse therapy with methylprednisolone (mPSL) 1000 mg/day for three consecutive days three times (days 9-11, 36-38, and 56-58); however, the disease was refractory, and the high proteinuria level was maintained. Thus, TAC was initiated on day 22, and on day 69, MT with MMF was initiated. For eight months of from day 69 to 309, the MMF dose was maintained between 0.5 and 1.0 g/day, with poor improvement in proteinuria (level > 2 g/g Cr). Further, it was difficult to reduce the PSL dose to <20 mg/day. Because proteinuria levels showed no remission, we considered this case as refractory LN. On day 310, the MMF dose was increased to 1.5 g/day without increasing steroid dose in MT, resulting in a reduction in the level of proteinuria to approximately 1 g/g Cr on day 366. Therefore, the PSL dose was reduced to 15 mg/day. On day 401, the MMF dose was increased to 1.75 g/day, and the PSL dose was reduced to 12.5 mg/day. The proteinuria level gradually declined, and complete remission occurred on day 429. On day 467, the MMF dose was further increased to 2.0 g/day, and the PSL dose was reduced to 10 mg/day. On day 964, the PSL dose was reduced to 6 mg/day. Thus, both induction and maintenance of complete remission were achieved. We believe that increasing the MMF dose in MT to over 1.5 g/day was effective. The time between the MMF dose increase to 1.5 g/day and complete remission was 119 days. No adverse events were observed.

In summary, complete remission was achieved in the two reported consecutive cases of refractory LN with conventional MT by gradually increasing the MMF dose in MT consisting of PSL+TAC+MMF without increasing steroid dose. Lymphopenia, hypogammaglobulinemia, gastrointestinal disturbances, or infections were not observed. No other adverse effects were observed. The increased dose of MMF that achieved complete remission within 6 months of administration is determined to be the most effective therapeutic dose of MMF. The effective doses of MMF as part of MT were 2.5 and 1.5 g/day for cases 1 and 2, respectively. The steroid dose was gradually tapered, as determined by the attending physician based on the disease activity, and TAC (3 and 2 mg/day for cases 1 and 2, respectively) was continuously administered without changing its dose. In this report, complete remission was defined as a proteinuria level < 0.5 g/g Cr with normal kidney function without any recurrence. Although refractory LN remains undefined; in this report, we defined it as any stage III or IV LN in which complete remission was not achieved with conventional MT in more than six months. Proteinuria, eGFR, PSL dose, and SLEDAI reflected LN activity, while peripheral lymphocyte counts and serum IgG values reflected an immune response. These parameters were assessed at the time when the most effective dose of MMF was reached and 1, 3, 6, 12, and 18 months after increasing MMF to its effective dose (Table 2). All parameters improved after 18 months, and it was possible to effectively reduce the PSL dose to 5.5 ± 1.5 mg/day. Moreover, lymphopenia and hypogammaglobulinemia were not observed, unlike the case when MMF was used at 1 g/day in MT. Lymphocytes and IgG significantly increased, providing protection to patients from infectious diseases to avoid further complications.

**Table 2:** Parameters change over 18 months after the MMF dose increase (1.5–2.5 g/day) eGFR: Estimated Glomerular Filtration Rate; MMF: Mycophenolate Mofetil; PSL: Prednisolone; SLEDAI: Systemic Lupus Erythematosus Disease Activity Index.

Parameters		No. of months after the MMF dose increment					
		0	1	3	6	12	18
Proteinuria (mg/g Cr)	Case 1 (MMF, 2.5 g/day)	613	630	545	366	156	110
	Case 2 (MMF, 1.5 g/day)	1600	1600	1040	340	110	80
eGFR (mL/min/1.73 m^2^)	Case 1 (MMF, 2.5 g/day)	79	84	89	89	88	92
	Case 2 (MMF, 1.5 g/day)	63	66	74	83	83	79
Peripheral lymphocyte count (/μL)	Case 1 (MMF, 2.5 g/day)	950	1400	1080	1800	1560	1320
	Case 2 (MMF, 1.5 g/day)	507	660	580	910	1210	1510
Serum IgG (mg/dL)	Case 1 (MMF, 2.5 g/day)	675	662	623	693	686	713
	Case 2 (MMF, 1.5 g/day)	346	417	432	510	563	672
PSL dose (mg/day)	Case 1 (MMF, 2.5 g/day)	12.5	12.5	12.0	10.0	7.0	4.0
	Case 2 (MMF, 1.5 g/day)	20.0	17.5	12.5	10.0	9.0	7.0
SLEDAI	Case 1 (MMF, 2.5 g/day)	4	4	4	0	0	0
	Case 2 (MMF, 1.5 g/day)	10	8	8	4	4	4

## Discussion

The incidence and severity of renal impairment are higher in Asian patients with SLE than Caucasian patients with SLE, with LN being one of the major causes of chronic kidney failure. To improve renal outcomes, it is important to formulate an optimal strategy for LN management [5]. When we utilized MT for the remission of refractory LN in Japanese patients, we increased the MMF dose without increasing steroid dose over the range stated in conventional protocols [11,13]. In conventional MT, the recommended MMF dose is 1.0 g/day [11,13]. Based on this recommendation, we initially treated the patients with an MMF dose of 1.0 g/day. A previous study involving 16 Japanese stage III-V LN patients reported that complete remission was achieved after 138 ± 114 days of MT with 43.4 ± 10 mg/day PSL, 935 ± 144 mg/day MMF, and 2.3 ± 0.8 mg/day TAC [13]. However, favorable therapeutic outcomes for our cases were not achieved despite a 6-month treatment with conventional MT. Additionally, proteinuria improvement was poor, therefore, we gradually increased the MMF dose to 1.5-2.5 g/day to achieve remission without increasing steroid dose since MMF has fewer adverse events than the steroid. Six months after the dose increase, complete remission was achieved in both the patients consecutively, who ultimately responded to the increased MMF dose without increasing the steroid dose. Active reductions in steroid doses were also effective; therefore, we believe that increasing the MMF dose played an important role in the outcomes observed.

The mean number of days from the start of the effective MMF dose (1.5-2.5 g/day) to complete remission was 129.5 ± 10.5 days. Thus, for cases that are difficult to treat using conventional MT, increasing MMF dose alone can induce complete remission, and minimizes the steroid use.

Previously, the recommended treatment regime for stage III and IV LN included early combined use of immunosuppressants and a reduction of steroids to a dose below 10 mg/day (PSL conversion) within 3 months [14]. The current therapeutic trend is the combination of immunosuppressants and the reduction of steroids to a maintenance level. Recently, the immunosuppressant CYC has been avoided because it may be teratogenic and carcinogenic. Therefore, MMF and TAC, which are both effective in inducing remission in LN, have been increasingly used. The increased doses of MMF without increasing steroid dose administered to the two patients allowed avoiding short- and long-term adverse events of steroids used in MT, which is consistent with the current policy that calls for lower doses of steroids [14]. In Japan, the TAC dose used to induce remission is normally 3 mg/day, and the dose is titrated to maintain the target concentration of 5 ng/mL at 12 h after dosing (C12). Although this dose has been established, MMF doses used to induce remission in practice vary considerably, from 1 to 3 g/day, also in Japan [13].

Different studies have reported the following optimal doses of MMF to induce LN remission: the European League Against Rheumatism (EULAR) reported a target dose of 3.0 g/day for stage III/IV cases; KDIGO reported that the maximum dose of MMF is 3.0 g/day for stage III/IV cases; and ACR reported a dose of 3.0 g/day for non-Asians and 2.0 g/day for Asians [1-2, 15]. Other recommendations for stage III to V cases include a target dose of 1.5 to 2.0 g/day, an initial-year dose of at least 1.5 g/day, and a second-year dose of at least 1.0 g/day [8,11]. These data indicate that the doses of MMF required to induce remission are lower in Asians than that in non-Asians [2].

However, it has been reported that 3.0 g/day is the preferable MMF dose for patients with stage III/IV crescentic LN and for those with proteinuria and significantly elevated Cr levels [2]. Thus, the optimal dose of MMF ranges from 1 to 3 g/day based on the physician’s judgment. The therapeutic outcomes achieved with MMF also differ depending on race and ethnicity [16].

The success rate of MMF administration to Chinese patients has been reported to be ≥80%, and the treatment has been reported to be highly effective and well-tolerated [8,11]. The results obtained in Chinese patients have led to a conclusion that the recommended MMF dose can be lowered. The currently listed clinical reference data for LN refer to Chinese patients, whereas a paucity of data exists on the optimal dose for other Asian populations, such as Japanese, Koreans, and Malaysians [4-5,8,11]. In previous studies, 1.0 g/day MMF was administered as part of a conventional MT for Asian patients [10-11]. However, some LN patients are refractory to conventional MT.

Some studies have measured the trough or peak serum levels of mycophenolic acid (MPA), an active metabolite of MMF, and have proposed that these levels are used as therapeutic indicators of LN [17]. However, currently available data do not justify drug concentration monitoring [2]. Since it also has been reported that the MPA area under the plasma concentration-time curve is associated with a single-nucleotide polymorphism in LN patients, differences in the effective MMF doses may be explained by the patient’s ethnicity [18]. In Japan, the optimal dose of MMF remains to be determined, although we did not measure the plasma concentration of MPA since the insurance adaptation outside as a limitation of this report; determining the optimal blood level of MPA in MT is critical.

Investigation of the adverse events associated with MMF has shown that although serious infections and hospital admissions are rare, some patients experience diarrhea [8]. Therefore, it is critical to focus on the possible presence of cytomegalovirus when MT is used [13]. In the two consecutive cases examined in this report, there were no adverse events reported to date, infectious disease, lymphopenia, or hypogammaglobulinemia when the MMF dose was increased to 1.5-2.5 g/day, demonstrating the safety of the drug. Infections were prevented through a relative decrease in the steroid dose, still achieving a complete remission of LN. However, the immune response observed after increasing the dose of MMF is important, and clinicians should monitor the serum IgG values and peripheral lymphocyte counts and need to closely monitor the risk of infectious diseases.

EULAR recommends that MMF should be administered as a remission-inducing therapy for a period of at least 3 years [15]. However, no data exist supporting MMF use for more than 3 years, and the long-term prognosis of MMF combination treatment remains unknown [19]. Therefore, careful research is required to determine the maximum duration of MT administration, the lowest possible maintenance dose of steroids, and the adverse events associated with immunosuppressive therapy. In any patient, it is preferable to attempt to reduce or discontinue a drug used in immunosuppressive therapy only after symptoms have been stabilized.

## Conclusions

MT is considered highly safe and is used to treat LN; however, some LN patients can be refractory to conventional MT. We report two consecutive cases of refractory LN with conventional MT, in which high doses of MMF were used as part of MT. By increasing MMF dose to 1.5-2.5 g/day, while maintaining the steroid dose, we have achieved complete remission. Therefore, increasing the dose of MMF is a potentially viable therapeutic option for Japanese LN patients who are refractory to conventional MT.
